# Enhanced efficacy of ALT-803, an IL-15-based superagonist complex, in combination with immune checkpoint inhibitors in an orthotopic muscle invasive bladder tumor model in mice

**DOI:** 10.1186/2051-1426-3-S2-P348

**Published:** 2015-11-04

**Authors:** Wenxin Xu, Karen Kage, Warren D Marcus, Robby Newman, Sarah Alter, Peter R Rhode, Hing C Wong

**Affiliations:** 1 Altor Bioscience, Miramar, FL, USA; 2 Altor BioScience Corporation, Miramar, FL, USA

## 

Immune checkpoint therapy, which can target regulatory pathways of T cells to enhance antitumor immune responses, has led to important clinical advances and provides novel strategies for combination immunotherapies against cancer. ALT-803 is an IL-15 superagonist complex capable of stimulating T cell and NK cell responses without triggering Treg activity. ALT-803 exhibits improved pharmacokinetics and lymphoid organ biodistribution compared to native IL-15, allowing this complex to have more potent efficacy against various hematologic and solid tumor models. Antibodies that block checkpoint inhibitor interactions, such as PD-1/PD-L1 or CTLA-4, have shown clinical efficacy against both melanoma and lung cancer, and may also be active in other tumor types, including bladder cancer. Here, we report that the combination of immune checkpoint blockers with ALT-803 has potent anti-tumor activity in C57BL/6 mice bearing syngenic orthotopic MB49-luc bladder tumors. These tumor cells were found to express CTLA-4 ligands and PD-L1 on the cell surface; thus, blockade of both CTLA-4 and PD-1/PD-L1 pathways were evaluated. Treatment of mice bearing established MB49luc bladder tumors with ALT-803 monotherapy exhibited a statistically significant increase in survival compared to controls (P < 0.05). However, the combination of ALT-803 with anti-PD-L1 and anti-CTLA-4 Abs further prolonged survival compared to control or related monotherapies. This effect was also seen with combination therapy of ALT-803+anti-PD-1 and ALT-803+anti-PD-1/anti-CTLA-4 Abs. Additionally, mice that were cured of MB49-luc tumors by ALT-803 plus anti-PD-L1/anti-CTLA-4 Ab therapy were resistant from bladder tumor rechallenge without further drug treatment whereas age-matched treatment-naïve mice developed tumors and die following MB49-luc tumor cell instillation. These results demonstrate that a short course of ALT-803 plus immune checkpoint inhibitor treatment not only has potent efficacy against established MB49luc tumor, but is also capable of inducing long-lasting protective immunologic memory against subsequent tumor cell rechallenge. Characterization of immune cell activity responsible for improved antitumor efficacy is underway. Overall, these results confirm that enhanced antitumor responses can be achieved by combining immune checkpoint blockers with the immunostimulatory agonist ALT-803, warranting evaluation of these strategies in the clinical setting.

